# Trend of Age-Adjusted Rates of Pediatric Traumatic Brain Injury in U.S. Emergency Departments from 2006 to 2013

**DOI:** 10.3390/ijerph15061171

**Published:** 2018-06-05

**Authors:** Cheng Chen, Jin Peng, Eric A. Sribnick, Motao Zhu, Henry Xiang

**Affiliations:** 1 Center for Pediatric Trauma Research, The Research Institute at Nationwide Children’s Hospital, Columbus, OH 43215, USA; chencheng616001@gmail.com (C.C.); Jin.Peng@nationwidechildrens.org (J.P.); Eric.Sribnick@nationwidechildrens.org (E.A.S.); 2 Center for Injury Research and Policy, The Research Institute at Nationwide Children’s Hospital, Columbus, OH 43215, USA; Motao.Zhu@nationwidechildrens.org; 3 College of Public Health, The Ohio State University, Columbus, OH 43210, USA; 4 Department of Pediatrics, The Ohio State University College of Medicine, Columbus, OH 43205, USA; 5 Department of Neurosurgery, Nationwide Children’s Hospital, Columbus, OH 43205, USA

**Keywords:** traumatic brain injury, pediatric, emergency department, annual percent change

## Abstract

*Objective*: To use the 2006–2013 Nationwide Emergency Department Sample (NEDS) database to describe trends of age-adjusted rates of pediatric traumatic brain injuries (TBI) treated in U.S. emergency departments. *Methods*: Time trend analysis was conducted on age-adjusted rates among children ≤17 years in the U.S. The annual percent change (APC) was calculated by fitting a least squares regression to the logarithm of the rates, using the calendar year as an independent variable. *Results*: In males, motor-vehicle-related trauma (APC −2.5%) and severe TBI (APC −3.6%) decreased over the study time period. Conversely, concussion (APC 5.1%), unspecified head injury (APC 6.6%), fall-related TBI (APC 7.1%), and mild TBI (APC 5.9%) increased. In females, severe TBI (APC −3.3%) decreased over the study time period and concussion (APC 6.5%), unspecified head injury (APC 7.2%), fall-related TBI (APC 7.6%), and mild TBI (APC 6.8%) increased. *Conclusion*: The overall age-adjusted rates of pediatric TBI-related emergency department (ED) visits increased from 2006 to 2013, which is largely caused by pediatric mild TBIs, especially unspecified injury to the head (ICD-9-CM code 959.01) and concussion. In comparison, age-adjusted rates of pediatric severe TBIs decreased. A major contributing factor might be a reduced number of traffic-related head trauma.

## 1. Introduction

Traumatic brain injury (TBI) is the leading cause of death and disability among children and adolescents in the U.S. [1] Each year, an estimated 50,000–60,000 U.S. children are hospitalized for TBI, at a rate of 70–75 cases per 100,000 children [2,3]. These injuries can have long-term physical and emotional outcomes that are difficult to detect; consequently, TBI not only impacts the life of a child and his/her family, but also has substantial medical costs [4,5]. Since the mid-1990s, laws, such as the Public Law 104–166 [6], the TBI Act Amendments of 2000 [7], and the TBI Act of 2008 [8], have guided federal agencies, researchers, and TBI prevention programs to develop and strengthen existing surveillance systems and prevention efforts to address the public health impact of TBIs. In 2008, the U.S. Congress authorized federal agencies, such as the Centers for Disease Control and Prevention (CDC), to engage in activities to decrease the severity and incidence of TBI [9]. These laws have prompted federal agencies and state health departments to target pediatric traumatic brain injuries as a public health priority. Furthermore, research findings about the negative impacts of sports-related traumatic brain injuries, including concussions, have raised awareness of TBIs and the long-term negative consequences among parents. However, a mixed trend of pediatric TBIs has been noticed during the past decade in the U.S.

Downward trends of TBI incidence in the U.S. are expected because of the prevention and educational efforts. However, based on the Nationwide Emergency Department Sample (NEDS) database, we found that emergency department (ED) visits for pediatric TBI (including the International Classification of Diseases, Ninth Revision, Clinical Modification (ICD-9-CM) 959.01) increased by 34.1% from 2006 to 2013 [10], exceeding the population growth of 6% over the same time period [11]. This increasing trend of TBI is consistent with Coronado, Marin, and Zonfrillo’s research findings [12,13,14], but two important issues remain unclear. First, we need a more in-depth understanding of the changes in different types of pediatric TBI-related ED visits, injury mechanisms, and severities to determine what factors are affecting the changing trends. While more concussion cases are seen at emergency departments or sports clinics, severe TBIs have a downward trend. Second, when comparing incidences from different years, we need to consider the variation of the population composition, so it is necessary to standardize the population composition for different years before analyzing the trend. Understanding these issues will not only provide important evidence about the trend of pediatric traumatic brain injuries, but also help guide future interventions in the U.S. This study investigates the epidemiological trends for pediatric TBI-related ED visits in the U.S. using statistical methods to account for these two issues. We extend previous research by addressing two unexamined questions: (1) Have age-adjusted rates of pediatric TBI-related ED visits been increasing since 2006 and, if so, by how much? and (2) Does the change in TBI-related visit rate vary across injury causes?

## 2. Materials and Methods

### 2.1. Data Sources

We used the 2006–2013 NEDS database, which is part of the Healthcare Cost and Utilization Project (HCUP) and is sponsored by the Agency for Healthcare Research and Quality (AHRQ). NEDS is the largest publicly available all-payer ED database in the U.S. [15]. Built on a 20-percent stratified sample of community, non-rehabilitation, and hospital-based EDs, NEDS contains data regarding approximately 30 million ED visits each year from more than 900 hospitals. National estimates can be calculated using the provided yearly weights.

We obtained population estimates from the U.S. bridged-race population estimates. The National Center for Health Statistics releases bridged-race population estimates from the July 1st resident population in the U.S. [16]. Between 1990 and 2015, postcensal estimates are available for the years 2010 and later, and intercensal estimates are available for the years 2000–2009.

### 2.2. Definition of Pediatric TBI

TBI cases were identified using the International Classification of Diseases, Ninth Revision, Clinical Modification (ICD-9-CM) diagnosis codes, recommended by the CDC for TBI surveillance [17]. All visits in the NEDS database were searched for ICD-9-CM codes for TBI. The CDC recommended TBI definition and TBI types include the following ICD-9-CM diagnosis codes: 800 (Fracture of vault of skull); 801 (Fracture of base of skull); 803 (Other and unqualified skull fractures); 804 (Multiple fractures involving skull or face with other bones); 850 (Concussion); 851 (Cerebral laceration and contusion); 852 (Subarachnoid, subdural, and extradural hemorrhage, following injury); 853 (Other and unspecified intracranial hemorrhage, following injury); 854 (Intracranial injury of other and unspecified nature); 950.1 (Injury to optic chiasm); 950.2 (Injury to optic pathways); 950.3 (Injury to visual cortex); 995.55 (Shaken infant syndrome); and 959.01 (Head injury, unspecified). TBI related ED visits were included if one or more of the 15 diagnosis fields contained a TBI ICD-9-CM diagnosis.

TBI injury mechanism was identified using the ICD-9 external-cause-of-injury codes. Codes were separated into four categories: Motor vehicle trauma (MVT) (motor vehicle traffic including occupant, motorcyclist, pedal cyclist, pedestrian, or unspecified), fall, struck by or against, and other [18].

### 2.3. Definition of Pediatric TBI Severity

We used a special STATA statistical software program: The ICD Programs for Injury Categorization (ICDPIC) (Version 3.0) to generate Abbreviated Injury Scale (AIS) scores for each TBI case [19]. TBI cases were defined as severe in patients with a head AIS score ≥3. Patients with an AIS score of 1 or 2 were defined as mild TBI cases and patients with a score of <1 were not further specified.

### 2.4. Statistical Analysis

Data analyses were conducted using the SAS Studio 3.4 Enterprise Edition (SAS Institute Inc., Cary, NC, USA). Estimates of TBI-related ED visits were weighted to represent all pediatric visits in community, non-rehabilitation, and hospital-based EDs in the U.S. The annual crude rates of TBI-related ED visits were estimated by dividing estimates by the total population in the U.S. during 2006–2013. Age-adjusted rates were calculated by the direct standardization method, using the U.S. population from the 2010 Census as the standard.

Time trend analysis was conducted on age-adjusted rates of each subgroup using the annual percent change (APC), which are assumed to change at a constant percentage of the rate of observed years. The APC is calculated by fitting a least squares regression line to the logarithm of the rates and using the calendar year as an independent variable [20,21,22,23]. Testing the hypothesis that the APC is equal to zero is equivalent to testing the hypothesis that the regression parameter is equal to zero [22,23]. Confidence intervals for the APC were also calculated.

## 3. Results

**Figure 1:**
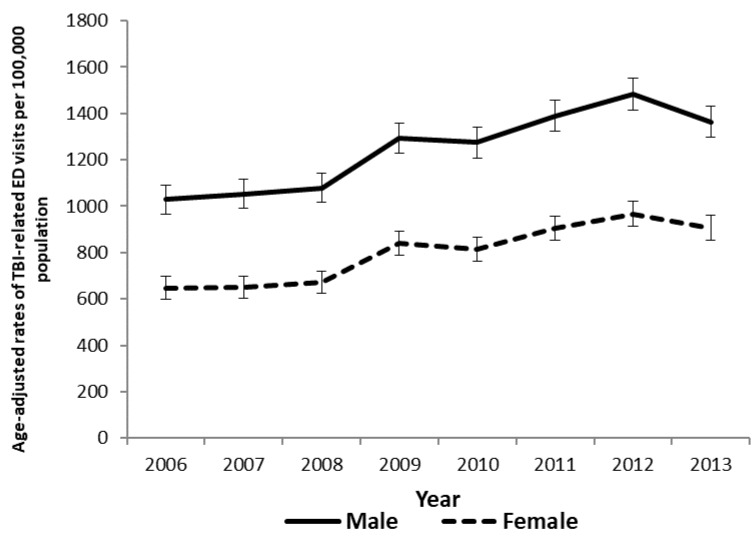
Age-adjusted rates of traumatic brain injury-related emergency department visits increased from 2006 to 2013 in both males and females in the U.S.

To illustrate national trends in the pediatric TBI incidence, we calculated crude rates of TBI-related ED visits by age and the age-adjusted rates of pediatric TBI related ED visits by sex (Table 1 and Figure 1). In different sexes and years, crude rates of TBI related ED visits began to decline from 0 to around 6 years of age and then increased to peak at age 16 or 17 (Table 1). The age-adjusted rates of pediatric TBI-related ED visits increased significantly from 1029.8 per 100,000 population in 2006 to 1364.3 per 100,000 population in 2013 for males (*p* < 0.05) and from 647.7 per 100,000 population in 2006 to 906.5 per 100,000 population in 2013 for females (*p* < 0.05).

In males, intracranial injury of other and unspecified nature (with APC −10.0%); cerebral laceration and contusion (APC −7.7%); fracture of base of skull (APC −3.3%); subarachnoid, subdural, and extradural hemorrhage, following injury (APC −2.3%); motor vehicle related trauma (APC −2.5%); and severe TBI (APC −3.6%) decreased significantly over the study time period (Table 2). In the meantime, concussion (APC 5.1%); unspecified head injury (APC 6.6%); fall related TBI (APC 7.1%); struck related TBI (APC 9.8%); and mild TBI (APC 5.9%) increased significantly (Table 2).

In females, intracranial injury of other and unspecified nature (APC −9.3%); cerebral laceration and contusion (APC −7.5%); shaken infant syndrome (APC −6.8%); fracture of base of skull (APC −2.5%); and severe TBI (APC −3.3%) decreased significantly over the study time period (Table 3). Meanwhile, concussion (APC 6.5%); unspecified head injury (APC 7.2%); fall related TBI (APC 7.6%); struck related TBI (APC 11.6%); and mild TBI (APC 6.8%) increased significantly. The age-adjusted rates of pediatric TBI-related ED visits in males and females for TBI types, injury mechanisms, and severities had the same trend.

## 4. Discussion

In the present study, the 2013 age-adjusted rates of pediatric TBI-related ED visits in males represent a 32.4% increase over the year 2006 and a similar increase was found in females. Other studies support our findings: The CDC reported the rates of TBI-related ED visits in youth, ages four years and younger, as increasing by more than 50% from 1374.0 to 2193.8 per 100,000 between 2007 and 2010 [1]. Furthermore, Coronado analyzed trends of TBI from 1995 to 2009 in the U.S. and found that the rate of TBI-related ED visits increased slowly from a rate of 434.1 in 1995 to 479.2 per 100,000 in 2006, with rates spiking sharply in 2008, then continuing to climb through 2009 to a rate of 686.0 per 100,000 [12]. Rates of pediatric TBI-related ED visits from various data sources all revealed a trend of rapid increase since 2006 [1,12].

TBI is a complicated condition, including both severe TBI, such as intracranial injury of other and unspecified nature and cerebral laceration and contusion, which impacts the life of an individual [1], and mild TBI, such as concussion, which are usually not life-threatening [1]. We need more in-depth understanding of the changes in different types of pediatric TBI-related ED visits, injury mechanisms, and severities to determine how current policies and interventions affect the changing trends. Our study provides evidence that the rates of pediatric traumatic brain injuries are still high and we have not seen a downward trend yet. These findings underscore the importance of continuous targeting of pediatric traumatic brain injuries as a top public health issue in the U.S.

In our research, concussion and unspecified head injury types, fall or struck injury mechanisms, and mild TBI severity showed a similar increasing trend. The reason for this similarity may be that most concussions and unspecified head injuries will be classified as mild TBI for males and females, and the highest annual percent changes are head injury-unspecified (ICD-9-CM code 959.01). Bazarian and colleagues examined the case definitions for TBI and found that the ‘unspecified’ ICD-9 codes made up 58% of the TBI cases, with 62.4% of these ‘unspecified’ cases being false positives [24]. Thurman concluded that estimates of the incidence of mild TBI treated in EDs, rather than being admitted, could be overestimated when using the ICD-9-CM code 959.01 in the U.S. [25]. With the exception of ‘unspecified injury to the head’ (code 959.01), the driving force of increasing rates of pediatric mild TBI-related ED visits is ‘concussion’, which dominantly contributed to increasing rates of TBI-related ED visits. Our findings are consistent with those of previous studies, which demonstrate the rates of referrals for pediatric concussions to neurologists have increased 150% in the 2011–2012 school year as compared to the 2008–2009 school year [26].

The increase in pediatric concussions may be due to the heightened awareness of the medical and public health issue, prompting more individuals with unspecified head injuries to seek medical care than before. In the 1990s, studies showed that, of the 1.5 million non-institutionalized U.S. civilians who had a self-reported TBI, 25% did not seek care [27]. Efforts from professional sports organizations [12], expanded media coverage about TBI among U.S. service members [28,29,30], and additional state legislation could all be factors contributing to this heightened awareness of TBI in youth [31], which in turn prompted more parents to send their children to emergency departments or sports clinics.

In addition to the factors that might have caused rates of TBI-related ED visits to rise, we found age-adjusted rates of severe pediatric TBI-related ED visits decreased in males and females. Particularly, intracranial injury of other and unspecified nature and cerebral laceration and contusion decreased with annual percent changes of about −10% and −7%, respectively. Based on the National Hospital Discharge Survey (2001–2010), rates of TBI-related hospitalizations in youth, age 5–14 years, dropped more than 50% from 2007–2010, decreasing from 54.5 to 23.1 hospitalizations per 100,000 U.S. population [32]. Similarly, Beck et al. found a significant decline in the incidence of severe TBI, moving from 5.0 to 3.2 cases per 100,000 population per year in Victoria, Australia from 2006 to 2014 [33]. The downward trend of pediatric severe TBI-related ED visits is apparent in our study. We also noticed that the age-adjusted rates of pediatric TBI-related ED visits caused by MVT decreased significantly in males in the present research. Coronado and Beck’s research also supports that the major factor contributing to a decline in severe TBI is due to a decrease in motor vehicle crashes [33,34]. These decreases in MVT have been attributed to the implementation of many federal and state regulations and traffic safety interventions, including the widespread use of seat belts, airbags, child safety seats, and motorcycle helmets [35,36]; the implementation of graduated licensing of new drivers and education programs to improve driver performance and safety [9,37]; and road safety engineering programs [38].

A major strength of this study is the ability to generate nationally representative estimates from the large number of ED records in the NEDS database. However, our research does have several limitations. First, the NEDS datasets do not have unique patient identifiers that allow us to identify and exclude individual patients who visited hospital EDs multiple times in one year. Thus, multiple records may exist in the dataset for a single patient. Second, we only used a single measure (head AIS) to identify the severity of head injuries due to the nature of the NEDS data. Other studies have identified injury severity using physiologic criteria, as well as required resources and interventions during trauma care [39,40]. Our rationale is that head AIS is a common criterion used by previous researchers for identifying severe TBIs and directly correlated with trauma patient mortality [10].

## 5. Conclusions

The age-adjusted rates of pediatric TBI-related ED visits increased from 2006 to 2013, which is largely caused by increases in pediatric mild TBI, especially unspecified injury to the head (code 959.01) and concussion. Conversely, age-adjusted rates of pediatric severe TBIs decreased and a major factor might be a reduced number of traffic-related head traumas. Our findings underscore the significance of pediatric TBIs in the U.S. and the need for national vigilance about this public health issue. The U.S. still needs to invest in pediatric TBI research and surveillance, and, most importantly, should develop effective interventions to slow down the upward trends of concussions among U.S. children. 

## Figures and Tables

**Table 1:** Crude rates of trauma brain injury (TBI)-related emergency department (ED) visits by age and sex in the U.S., 2006–2013.

Age	(Rate/100,000)															
	Male								Female							
	2006	2007	2008	2009	2010	2011	2012	2013	2006	2007	2008	2009	2010	2011	2012	2013
0	371.0	409.2	421.4	412.8	405.8	415.0	413.4	387.8	288.7	321.8	329.7	334.7	327.8	325.9	313.8	310.5
1	278.2	306.3	323.7	322.7	350.8	314.3	307.4	279.1	249.0	251.5	280.2	286.2	283.2	269.5	258.5	249.4
2	261.5	275.3	288.5	293.5	308.7	296.9	274.4	261.1	198.6	227.8	234.4	237.3	253.4	234.3	238.6	204.2
3	225.6	225.5	237.1	268.0	270.7	253.5	250.4	228.3	165.7	177.5	189.2	193.9	191.8	173.4	204.6	181.5
4	190.9	196.3	222.4	222.6	232.1	207.4	230.2	218.1	136.9	142.3	154.6	150.3	155.4	148.8	166.3	154.7
5	198.1	215.4	217.5	217.9	234.6	219.5	235.8	224.5	129.7	133.8	141.1	128.1	128.9	141.6	155.4	145.6
6	211.0	213.0	213.6	232.5	234.6	222.5	241.7	247.2	126.0	124.4	128.9	147.0	145.2	134.6	149.0	145.2
7	216.9	217.6	220.8	239.8	236.4	243.5	255.2	252.9	134.5	113.9	122.3	134.3	128.5	137.2	138.4	144.2
8	221.3	234.0	245.1	236.0	244.1	247.5	284.7	280.1	120.9	129.8	125.1	135.8	137.8	122.4	140.3	135.9
9	225.4	247.1	268.2	278.5	265.6	288.3	331.5	335.2	120.9	133.3	121.5	126.3	127.2	137.6	150.7	141.7
10	265.8	273.4	282.2	310.1	315.0	332.3	395.3	376.5	121.0	118.5	121.8	129.2	145.6	134.4	155.1	161.6
11	299.0	338.7	329.3	367.1	392.1	430.0	473.0	470.3	126.2	126.4	132.5	149.4	157.8	155.8	182.7	184.8
12	352.0	386.0	402.1	431.2	480.9	480.3	526.8	536.2	152.2	158.0	154.8	185.7	192.4	202.0	244.3	254.1
13	437.0	455.0	489.3	498.9	553.0	619.7	675.1	636.7	195.4	191.0	202.4	241.4	240.1	277.2	313.5	331.4
14	570.6	563.2	628.2	629.2	719.4	726.7	785.0	746.5	256.8	245.4	274.4	311.2	348.1	392.0	443.1	450.9
15	651.5	668.2	691.3	741.1	758.0	797.6	853.6	792.2	301.5	302.3	314.6	358.2	387.4	436.3	497.1	475.9
16	742.7	737.9	719.7	778.7	807.5	802.2	858.8	801.7	348.1	361.3	377.0	405.0	414.9	443.3	486.6	469.8
17	775.7	767.6	789.6	808.1	859.7	775.0	808.6	771.9	387.2	358.8	391.4	401.7	429.0	434.6	463.5	471.5

**Table 2:** Trend of age-adjusted rates of TBI-related ED visit by TBI type, injury mechanism, and severity in the U.S., 2006–2013, Males.

TBI Types/Reason	(Rate/100,000)								Annual Percent Change	(95% CI)		*p*-Value
	2006	2007	2008	2009	2010	2011	2012	2013				
**TBI type ^a^:**												
Intracranial injury of other and unspecified nature	16.7	14.7	11.8	11.1	11.7	9.0	7.5	8.5	−10.0	−13.2	−6.7	0.00
Cerebral laceration and contusion	7.1	7.7	6.2	6.1	5.8	5.1	4.7	4.3	−7.7	−9.6	−5.7	<0.0001
Shaken infant syndrome	0.8	1.0	0.8	0.5	0.6	0.7	0.6	0.7	−5.2	−11.6	1.7	0.11
Multiple fractures involving skull or face with other bones	0.6	0.6	0.5	0.7	0.5	0.5	0.5	0.4	−4.7	−10.3	1.3	0.10
Other and unspecified intracranial hemorrhage following injury	3.3	2.9	3.5	3.9	3.1	2.9	2.4	2.8	−3.4	−7.9	1.3	0.13
Fracture of base of skull	26.7	26.9	27.7	25.2	26.6	23.3	22.4	21.6	−3.3	−5.1	−1.6	0.00
Subarachnoid, subdural, and extradural hemorrhage, following injury	11.6	11.1	12.2	11.3	10.8	10.2	9.8	10.5	−2.3	−4.0	−0.5	0.02
Fracture of vault of skull	17.9	19.9	20.4	17.2	17.6	17.6	17.2	17.7	−1.5	−3.7	0.9	0.17
Other and unqualified skull fractures	8.4	8.1	8.4	8.2	9.1	8.6	8.4	8.2	0.3	−1.2	1.7	0.69
Concussion	271.8	284.7	300.8	324.9	344.8	352.6	386.7	365.6	5.1	3.7	6.5	<0.0001
Head injury, unspecified	664.9	675.0	686.5	884.7	844.0	959.7	1022.7	924.0	6.6	3.4	9.8	0.00
Injury to optic chiasm	0.0	0.1	0.0	0.0	0.1	0.1	0.2	0.2	21.2	−13.1	68.9	0.21
**TBI injury mechanism:**												
Motor Vehicle Trauma ^b^	93.4	89.2	82.3	82.4	84.4	78.2	81.3	75.1	−2.5	−3.7	−1.3	0.00
Other	246.1	233.1	244.9	221.5	194.3	220.8	209.5	229.3	−1.7	−4.4	1.0	0.17
Fall	423.0	457.4	470.8	590.8	589.7	644.3	698.6	620.8	7.1	3.9	10.3	0.00
Struck	267.3	273.1	280.7	399.2	406.2	446.9	493.7	439.1	9.8	5.4	14.3	0.00
**TBI severity ^c^:**												
Severe	60.1	61.6	61.6	56.2	56.6	51.6	48.5	49.1	−3.6	−5.0	−2.3	0.00
Not further specified (NFS)	0.5	0.6	0.4	0.4	0.4	0.5	0.4	0.6	−1.8	−8.2	4.9	0.52
Mild	969.2	990.6	1016.7	1237.3	1217.6	1338.2	1434.2	1314.6	5.9	3.4	8.4	0.00

^a^: TBI type were defined as if the first ICD-9-CM diagnosis code fell within the Centers for Disease Control and Prevention TBI definition, regardless of other diagnoses codes; ^b^: Injury by motor vehicle traffic, including the occupant of a car, motorcyclist, pedal cyclist, pedestrian, or unspecified person; ^c^: Severe: Head Abbreviated Injury Scale (AIS) ≥ 3; Mild: Head AIS = 1 or 2; Not further specified (NFS): No score

**Table 3:** Trend of age-adjusted rates of TBI-related ED visits by TBI type, injury mechanism, and severity in the U.S., 2006–2013, Females.

TBI Type/Reason	(Rate/100,000)								APC	(95% CI)		*p*-Value
	2006	2007	2008	2009	2010	2011	2012	2013				
**TBI type ^a^:**												
Intracranial injury of other and unspecified nature	10.4	9.0	7.5	6.6	7.0	5.1	5.0	5.7	−9.3	−13.0	−5.5	0.00
Cerebral laceration and contusion	3.7	4.3	3.3	3.2	3.0	2.3	2.4	2.6	−7.5	−11.1	−3.7	0.00
Shaken infant syndrome	0.6	0.7	0.6	0.6	0.7	0.4	0.4	0.4	−6.8	−11.7	−1.7	0.02
Multiple fractures involving skull or face with other bones	0.2	0.4	0.3	0.3	0.2	0.2	0.3	0.2	−5.7	−14.2	3.5	0.17
Other and unspecified intracranial hemorrhage following injury	1.8	1.9	1.8	1.8	1.5	1.9	1.2	1.5	−4.0	−8.3	0.4	0.07
Fracture of base of skull	6.7	6.0	6.4	5.8	5.5	5.3	6.0	5.4	−2.5	−4.5	−0.4	0.03
Subarachnoid, subdural, and extradural hemorrhage, following injury	12.2	13.3	13.7	12.5	12.8	10.6	11.0	11.5	−2.5	−5.1	0.2	0.07
Fracture of vault of skull	10.0	12.1	13.0	10.6	11.4	11.0	11.1	10.7	−0.4	−3.6	2.9	0.76
Injury to optic pathways	4.8	5.7	5.0	5.1	4.6	5.4	5.5	4.7	−0.1	−3.2	3.0	0.92
Concussion	148.3	148.5	160.1	179.8	187.5	198.1	219.9	215.3	6.5	5.1	7.8	<0.0001
Head injury, unspecified	449.0	448.9	461.2	612.5	580.2	664.2	702.9	648.5	7.2	3.9	10.7	0.00
Injury to optic chiasm	0.0	0.1	0.1	0.1	0.1	0.1	0.1	0.1	13.6	−2.1	31.7	0.08
**TBI injury mechanism:**												
Motor Vehicle Trauma ^b^	85.6	77.9	70.9	75.3	77.5	77.4	78.3	74.8	−0.7	−2.7	1.3	0.41
Other	126.7	119.5	126.1	124.2	104.1	122.7	117.4	133.9	0.0	−2.9	3.1	0.97
Fall	309.8	325.0	338.6	437.6	434.9	478.8	512.5	465.8	7.6	4.3	11.0	0.00
Struck	125.6	128.4	137.4	201.7	198.0	225.7	257.6	232.0	11.6	7.1	16.4	0.00
**TBI severity ^c^:**												
Not further specified (NFS)	0.4	0.5	0.3	0.4	0.4	0.3	0.3	0.3	−3.5	−7.9	1.1	0.11
Severe	30.6	31.9	32.1	28.8	28.5	25.2	26.2	26.2	−3.3	−5.1	−1.5	0.00
Mild	616.7	618.3	640.5	809.6	785.6	879.1	939.3	880.1	6.8	4.0	9.6	0.00

^a^: TBI type were defined as if the first ICD-9-CM diagnosis code fell within the CDC TBI definition, regardless of other diagnoses codes; ^b^: Including the occupant of a car, motorcyclist, pedal cyclist, pedestrian, or unspecified person; ^c^: Severe: Head AIS ≥3; Mild: Head AIS= 1 or 2; Not further specified (NFS): No score
